# Chemoprotective Mechanism of Sodium Thiosulfate Against Cisplatin-Induced Nephrotoxicity Is via Renal Hydrogen Sulfide, Arginine/cAMP and NO/cGMP Signaling Pathways

**DOI:** 10.3390/ijms26010384

**Published:** 2025-01-04

**Authors:** George J. Dugbartey, Karl K. Alornyo, Ismaila Adams, Samuel Adjei, Daniel Amoah, Richard Obeng-Kyeremeh

**Affiliations:** 1 Department of Pharmacology and Toxicology, School of Pharmacy, College of Health Sciences, University of Ghana, Accra P.O. Box LG43, Ghana; 2 Department of Physiology and Pharmacology, Accra College of Medicine, Accra P.O. Box CT9828, Ghana; 3 Department of Surgery, Division of Urology, London Health Sciences Center, Western University, London, ON N6A 5C1, Canada; 4 Matthew Mailing Center for Translational Transplant Studies, London Health Sciences Center, Western University, London, ON N6A 5C1, Canada; 5 Department of Medical Pharmacology, University of Ghana Medical School, Accra P.O. Box KB52, Ghana; 6 Department of Animal Experimentation, Noguchi Memorial Institute for Medical Research, College of Health Sciences, University of Ghana, Accra P.O. Box LG581, Ghana

**Keywords:** Cisplatin-induced nephrotoxicity (CIN), sodium thiosulfate (STS), hydrogen sulfide (H_2_S), propargylglycine (PAG), chemoprotection, nitric oxide (NO), arginine, cyclic adenosine monophosphate (cAMP), cyclic guanosine monophosphate (cGMP)

## Abstract

Cisplatin is a common and highly effective chemotherapeutic agent whose nephrotoxic side effect is well-characterized. Sodium thiosulfate (STS), an FDA-approved hydrogen sulfide (H_2_S) donor drug, is emerging as a chemoprotective agent against cisplatin-induced nephrotoxicity (CIN). In this study, we investigated the chemoprotective mechanism of STS in a rat model of CIN. Twenty-five male Sprague Dawley rats were randomly assigned to the following groups: HC: Healthy control (received 10 mL/kg/day of 0.9% saline intraperitoneally (*ip*), [n = 5]), CIN: Cisplatin (received single dose of 7 mg/kg cisplatin *ip* [n = 5]); CIN + PAG: Cisplatin and daily *ip* administration of 40 mg/kg of the H_2_S inhibitor, DL-propargylglycine (PAG) for 28 days (n = 5); CIN + PAG + STS: Cisplatin and daily PAG and STS (150 µM) *ip* injection for 28 days; CIN + STS: Cisplatin and daily STS *ip* administration for 28 days (n = 5). Rats in each group were kept in metabolic cages for 24 h on day 0, 14 and 29 after cisplatin administration for urine collection. Rats were then euthanized, and kidney and blood samples were collected for analysis. Histologically, CIN was characterized by glomerular and tubular injury and significant macrophage influx and tubular apoptosis, as well as markedly increased levels of plasma and renal IL-1β, IL-6 and TNF-α and impaired renal antioxidant status compared to HC rats (*p* < 0.001). These pathological changes were exacerbated in CIN + PAG rats and were strongly reduced in CIN + PAG + STS rats relative to CIN + PAG rats (*p* < 0.01), while superior renal protection was observed in CIN + STS rats. Functionally, CIN was evidenced by markedly increased levels of serum creatinine and BUN, and significantly decreased urine creatinine, renal creatinine clearance, as well as electrolyte imbalance and urinary concentrating defect in comparison with HC (*p* < 0.01). These functional changes worsened significantly in CIN + PAG rats (*p* < 0.05) but improved in CIN + PAG + STS rats, with further improvement in CIN + STS rats to levels comparable to HC rats. Mechanistically, STS increased renal and plasma levels of H_2_S, arginine, cAMP, nitric oxide (NO) and cGMP as well as SIRT3 and PGC-1α. We have shown for the first time that STS provides chemoprotection against CIN by activating renal arginine/cAMP and NO/cGMP signaling pathways and their downstream mechanisms through increased renal H_2_S production.

## 1. Introduction

Following the discovery of its anticancer activity in animal models in 1969 [1], cisplatin has become one of the most effective drugs widely used for the treatment of a variety of cancers, including ovarian, testicular, bladder, head and neck, and uterine carcinomas [2,3,4]. However, its chemotherapeutic use is limited by a well-established nephrotoxic side effect, which is observed following 10 days of cisplatin chemotherapy, and thus reduces the quality of life of patients [5,6,7]. In addition, dose adjustment or withdrawing cisplatin administration weakens the treatment effect. Despite administration of hypertonic saline, diuretics and aggressive hydration therapy to protect against renal complications following cisplatin administration, cisplatin-induced nephrotoxicity (CIN) has been reported in 30% of adult patients under high-dose cisplatin therapy [8,9] and 77% of adult patients receiving continuous low-dose cisplatin [10], while over 70% of pediatric patients also experience CIN [11]. Functionally, CIN is characterized by increased levels of serum creatinine and urea, along with electrolyte imbalance [12]. Additionally, renal vasoconstriction, increased renovascular resistance, decline in renal blood flow, reduced single-nephron glomerular filtration rate, decreased renal clearance in a dose- and time-dependent manner, and eventual loss of renal function also characterize CIN [5,13,14,15]. Histologically, cisplatin causes thickening of the glomerular basement membrane, proliferation of capsular epithelial cells, and injury to the glomerular capillaries and renal tubules [16,17,18,19].

While the mechanisms underlying CIN are not fully elucidated, epithelial cells in the S3 segment of the renal proximal tubule accumulate substantial concentrations of cisplatin due to upregulated expression of organic cation transporter-2 and high affinity copper transporter-1 in the inner cortex and outer medulla of the renal proximal tubules, which transport cisplatin into proximal tubular epithelial cells, thereby contributing to uneven concentration of cisplatin in the kidney tissue, and this subsequently impairs the reabsorption function of the renal tubules [19,20,21]. In fact, this also accounts for the five-fold increase in the concentration of cisplatin in renal proximal tubular epithelial cells compared to its concentration in serum [22]. In addition to the disproportionate concentration of cisplatin in the kidney tissue, a pathological pathway underlying CIN has been identified in the renal tubule. This includes (i) bioconversion of cisplatin into nephrotoxic metabolites, (ii) formation of DNA adducts, (iii) ROS-induced oxidative stress and mitochondrial dysfunction, (iv) induction of inflammation, and (v) activation of apoptotic machinery. Despite the identification of this pathological pathway, effective chemoprotection against CIN remains lacking. Therefore, targeting these and other injury mechanisms with novel and promising chemoprotective agents is essential in preventing or ameliorating CIN.

Hydrogen sulfide (H_2_S), the third established member of a family of gaseous signaling molecules, has been identified as a novel chemoprotective agent against CIN [23,24]. H_2_S is endogenously produced in all mammalian cells by two cytosolic enzymes, cystathionine-β-synthase (CBS) and cystathionine-γ-lyase (CSE), as well as the mitochondrial enzyme, 3-mecaptopyruvate sulfur-transferase (3-MST) and the peroxisomal enzyme, D-amino acid oxidase (DAO) [25,26,27]. While the distribution of these enzymes is subcellular and tissue-specific, they are abundantly expressed in the kidney, mainly localized in the S3 segment, the same region of the renal proximal tubules known for cisplatin accumulation [27,28,29]. In various animal models of CIN in which H_2_S was protective against CIN, H_2_S was administered via donor compounds such as sodium hydrosulfide (NaHS), GYY4137, and natural sources of H_2_S such as garlic-derived diallyl trisulfide, diallyl disulfide and diallyl sulfide [30,31,32]. However, these H_2_S donor compounds are not clinically viable, making the translation of their therapeutic efficacy in animal models of CIN difficult. This has encouraged the need for clinically viable H_2_S donors for CIN. Sodium thiosulfate (STS), an FDA-approved H_2_S donor drug, is emerging as a clinical drug with chemoprotective properties against CIN. Also, arginine/cyclic adenosine monophosphate (cAMP) and nitric oxide (NO)/cyclic guanosine monophosphate (cGMP) are important regulators of renal function and counteract pathophysiological conditions to preserve renal integrity in different kidney diseases. However, it is unknown whether STS influences arginine/cAMP and NO/cGMP in CIN. Therefore, the present study seeks to investigate the chemoprotective effect of STS and its influence on arginine/cAMP and NO/cGMP signaling pathways and downstream mechanisms in a rat model of CIN.

## 2. Results

### 2.1. STS Therapy Provides Chemoprotection by Preserving Renal Structural Integrity After Cisplatin Administration

**Figure 1:**
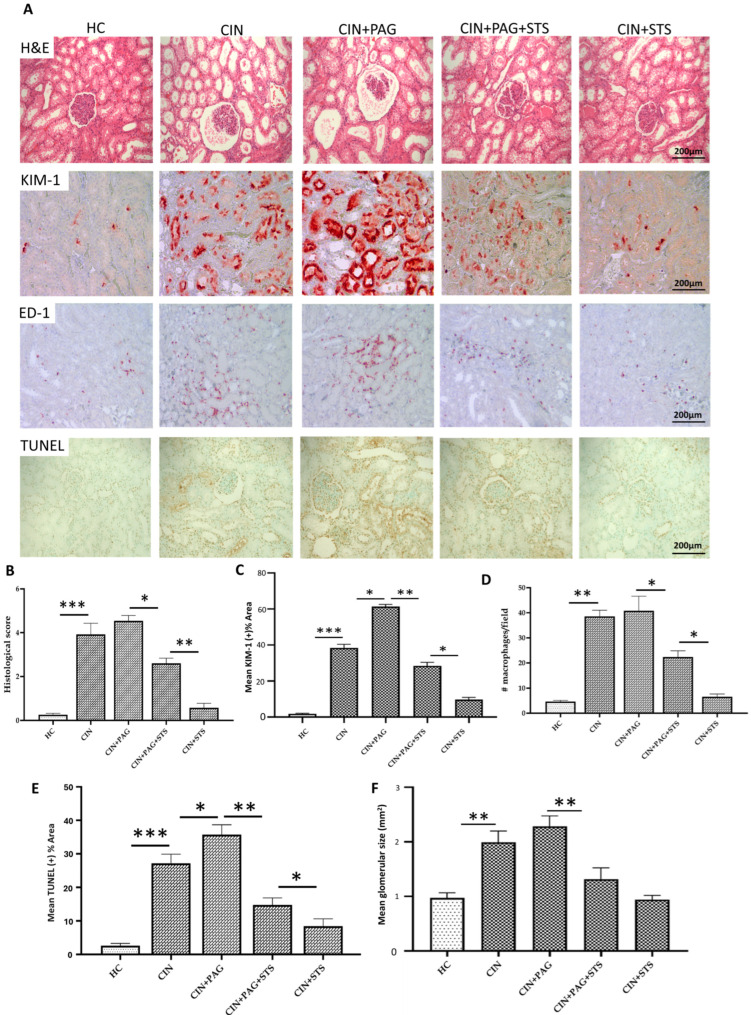
Histopathology and quantification of kidney tissue sections. (**A**) Representative images of kidney sections showing H and E, KIM-1, ED-1 and TUNEL stains. (**B**–**F**) Quantification of immune (histochemical) staining of (**B**) H and E, (**C**) KIM-1, (**D**) ED-1, (**E**) TUNEL and (**F**) glomerular size. HC = Healthy control group; CIN = Cisplatin group; CIN + PAG = Cisplatin group treated with propargylglycine; CIN + PAG + STS = Cisplatin group treated with propargylglycine and sodium thiosulfate; CIN + STS = Cisplatin group treated with sodium thiosulfate. All images were taken at 40× magnification. Values are mean ± SEM. * *p* < 0.05, ** *p* < 0.01, *** *p* < 0.001.

We examined the effect of cisplatin on renal morphology and STS chemoprotection by performing histology. Compared to kidneys of healthy control (HC) rats, cisplatin administration to rats in cisplatin (CIN) group caused significant glomerular and tubular injury as well as glomerular hypertrophy (Figure 1A,B,F; *p* < 0.001). Pharmacological inhibition of H_2_S with DL-propargylglycine (PAG) in cisplatin-treated rats (CIN + PAG) caused further injury to the glomerular and tubular compartments of the kidney in comparison with kidneys of rats in the CIN group (Figure 1A,B,F), while administration of STS to rats in the CIN + PAG group markedly reduced the injury relative to kidneys of rats in the CIN + PAG group (Figure 1A,B,F; *p* < 0.05). In the absence of PAG, however, STS administration to CIN rats preserved renal structural integrity to a level comparable to kidneys from HC rats (Figure 1A,B,F; *p* > 0.05). To determine the degree of renal injury caused by cisplatin and the chemoprotective effect of STS, we performed immunohistochemical staining for KIM-1 (tubular injury), ED-1 (macrophage infiltration) and TUNEL (apoptosis). Renal KIM-1 was significantly expressed in CIN rats relative to HC rats (Figure 1A,C; *p* < 0.001), while the expression was further increased in PAG-treated CIN rats (Figure 1A,C; *p* < 0.05). Although STS in PAG-treated CIN rats afforded substantial renal protection relative to CIN + PAG group (Figure 1A,C; *p* < 0.01), STS administration provided superior protection in the kidneys of CIN rats, which is comparable to kidneys of HC rats (Figure 1A,C; *p* > 0.05). The same trend was observed in ED-1 and TUNEL stain (Figure 1A,D,E). In summary, STS therapy protects and preserves renal structural integrity against development of CIN.

### 2.2. STS Therapy Offers Chemoprotection by Improving Body Weight and Renal Function Following Cisplatin Administration

**Figure 2:**
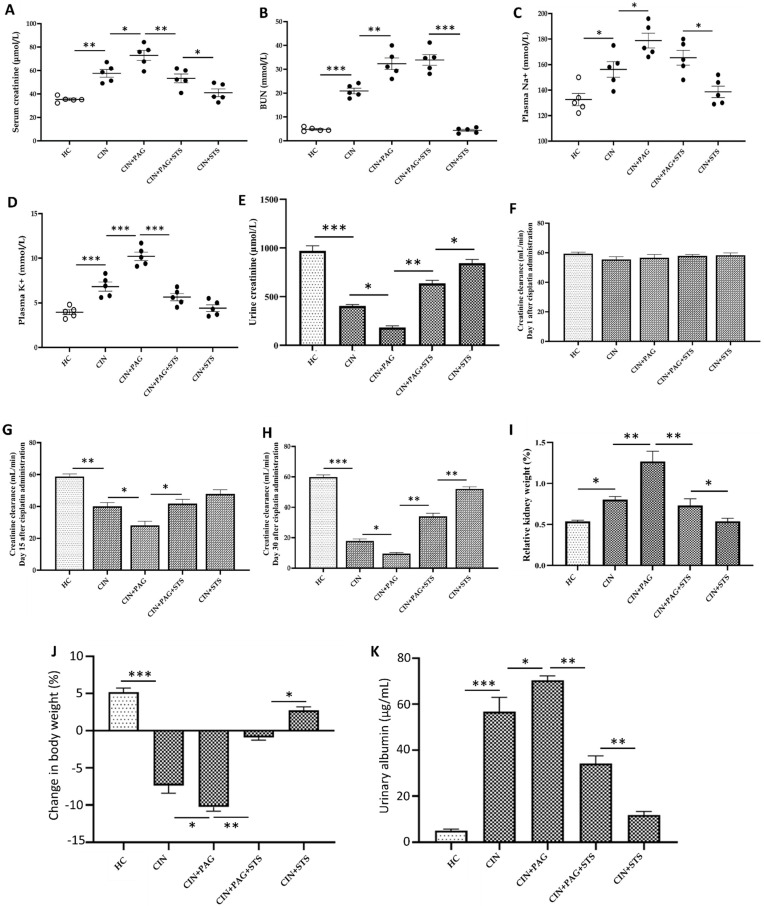
Parameters of kidney function. Levels of (**A**) serum creatinine, (**B**) blood urea nitrogen (BUN), (**C**) plasma Na^+^, (**D**) plasma K^+^, (**E**) urine creatinine, (**F**) creatinine clearance on day 1 after cisplatin administration, (**G**) creatinine clearance on day 15 after cisplatin administration, (**H**) creatinine clearance on day 30 after cisplatin administration, (**I**) relative kidney weight, (**J**) change in body weight, and (**K**) urinary albumin. HC = Healthy control group; CIN = Cisplatin group; CIN + PAG = Cisplatin group treated with propargylglycine; CIN + PAG + STS = Cisplatin group treated with propargylglycine and sodium thiosulfate; CIN + STS = Cisplatin group treated with sodium thiosulfate. All images were taken at 40× magnification. Values are mean ± SEM. * *p* < 0.05, ** *p* < 0.01, *** *p* < 0.001.

In addition to histopathological changes, we also measured markers of renal function. Administration of cisplatin elevated serum creatinine and blood urea nitrogen (BUN) levels significantly relative to HC rats (Figure 2A,B; *p* < 0.01), with additional increases in CIN + PAG rats compared to CIN group (Figure 2A,B; *p* < 0.05). Whereas serum creatinine level in CIN + PAG + STS group markedly decreased compared to the level in CIN + PAG rats (Figure 2A; *p* < 0.01), no significant changes in BUN level were observed between CIN + PAG and CIN + PAG + STS rats (Figure 2B; *p* > 0.05). Remarkably, serum creatinine and BUN levels in CIN + STS rats dropped significantly to the levels in HC rats (Figure 2A,B; *p* > 0.05). The same trend was observed in plasma Na^+^ and K^+^ levels, as well as in urine creatinine (Figure 2C–E).

**Figure 3:**
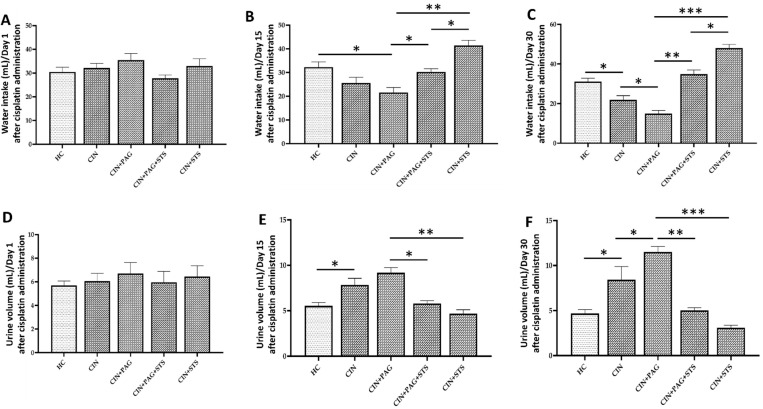
Water intake and urine output. Volumes of (**A**) water intake on day 1 after cisplatin administration, (**B**) water intake on day 15 after cisplatin administration, (**C**) water intake on day 30 after cisplatin administration, (**D**) urine output on day 1 after cisplatin administration, (**E**) urine output on day 15 after cisplatin administration, and (**F**) urine output on day 30 after cisplatin administration. HC = Healthy control group; CIN = Cisplatin group; CIN + PAG = Cisplatin group treated with propargylglycine; CIN + PAG + STS = Cisplatin group treated with propargylglycine and sodium thiosulfate; CIN + STS = Cisplatin group treated with sodium thiosulfate. All images were taken at 40× magnification. Values are mean ± SEM. * *p* < 0.05, ** *p* < 0.01, *** *p* < 0.001.

In addition, renal creatinine clearance was unchanged in all groups of rats on day 1 after cisplatin administration (Figure 2F). However, on days 15 and 30 after cisplatin administration, renal creatinine clearance decreased significantly in CIN rats compared to rats in HC group (Figure 2G,H; *p* < 0.01), with a further decrease in CIN + PAG rats in comparison with CIN group (Figure 2G,H; *p* < 0.05). Renal creatinine clearance on days 15 and 30 after cisplatin administration markedly increased in CIN + PAG + STS rats relative to CIN + PAG group (Figure 2G,H; *p* < 0.05), with an additional increase in CIN + STS rats to levels comparable to HC level, and negatively correlated with relative kidney weight (Figure 2G–I; *p* > 0.05). Interestingly, the trend in renal creatinine clearance is the same in water intake, with significant increases in CIN + STS rats on days 15 and 30 after cisplatin administration compared to HC rats (Figure 3A–C). However, the reverse was observed in urine volume on days 15 and 30 after cisplatin administration (Figure 3D–F). Moreover, cisplatin administration markedly decreased rat body weight and also caused significant proteinuria, which became worse in CIN + PAG rats compared to HC rats (Figure 2J,K; *p* < 0.001). However, STS treatment in CIN + PAG rats reversed these changes and produced a superior therapeutic efficacy in CIN group of rats to levels comparable to HC rats (Figure 2J,K). Taken together, STS therapy provides chemoprotection and body weight and improves renal function after cisplatin administration.

### 2.3. STS Therapy Affords Chemoprotection by Suppressing Renal Inflammation and Improving Renal Antioxidant Status After Cisplatin Administration

**Figure 4:**
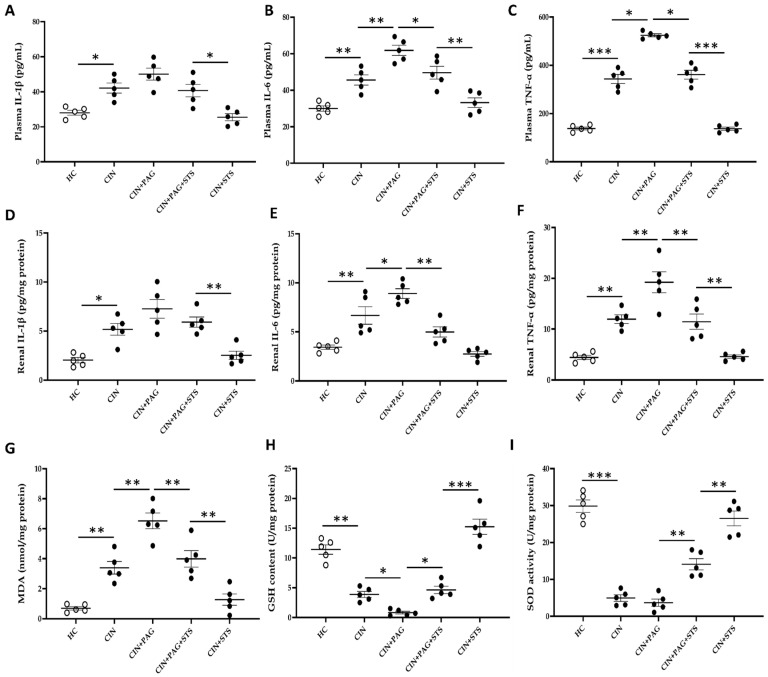
Anti-inflammatory and antioxidant effects of STS. Levels of (**A**) plasma interleukin-1beta (IL-1β), (**B**) plasma interleukin-6 (IL-6), (**C**) plasma tumor necrosis factor-alpha (TNF-α), (**D**) renal IL-1β, (**E**) renal IL-6, (**F**) renal TNF-α, (**G**) renal malondialdehyde (MDA), (**H**) renal glutathione (GSH), and (**I**) renal superoxide dismutase (SOD). HC = Healthy control group; CIN = Cisplatin group; CIN + PAG = Cisplatin group treated with propargylglycine; CIN + PAG + STS = Cisplatin group treated with propargylglycine and sodium thiosulfate; CIN + STS = Cisplatin group treated with sodium thiosulfate. All images were taken at 40× magnification. Values are mean ± SEM. * *p* < 0.05, ** *p* < 0.01, *** *p* < 0.001.

We also measured inflammatory and antioxidant status to determine their role in renal injury after cisplatin administration, and the effect of STS therapy. Cisplatin caused a substantial increase in plasma levels of IL-1β, IL-6 and TNF-α in comparison with HC rats (Figure 4A–C; *p* < 0.05). Administration of PAG to CIN rats raised the levels of plasma IL-6 and TNF-α significantly relative to rats in CIN group (Figure 4B,C; *p* < 0.05), with a slight increase in plasma IL-1β level compared to CIN rats (Figure 4A; *p* > 0.05). However, administration of STS to PAG-treated CIN rats markedly reduced the levels of plasma IL-6 and TNF-α in comparison with rats in CIN + PAG group (Figure 4B,C; *p* < 0.05) without significantly altering plasma IL-1β level compared to CIN + PAG rats (Figure 4A; *p* > 0.05). Interestingly, STS administration to CIN rats strongly decreased plasma levels of all three pro-inflammatory cytokines to HC level (Figure 4A–C; *p* > 0.05). The same trend was observed in renal IL-1β, IL-6 and TNF-α levels (Figure 4D–F).

Cisplatin also impacted renal antioxidant status by significantly increasing renal malondialdehyde (MDA) level relative to the level in HC rats (Figure 4G; *p* < 0.01), with a further increase in CIN + PAG rats compared to CIN rats (Figure 4G; *p* < 0.01). However, renal MDA level was markedly reduced in CIN + PAG + STS group of rats in comparison with CIN + PAG group (Figure 4G; *p* < 0.01). Remarkably, renal MDA level in CIN + STS rats decreased significantly to HC level (Figure 4G, *p* > 0.01). Additionally, cisplatin significantly decreased renal glutathione (GSH) content and superoxide dismutase (SOD) activity compared to HC rats (Figure 4H,I; *p* < 0.01), with additional decrease in CIN + PAG group in comparison with CIN rats (Figure 4H; *p* < 0.05). Interestingly, renal GSH content and SOD activity markedly increased in CIN + PAG + STS group relative to CIN + PAG rats (Figure 4H,I; *p* < 0.05), with substantial increases at HC level (Figure 4I; *p* > 0.05) and above HC level (Figure 4H; *p* < 0.05). Collectively, STS therapy improves renal inflammation and antioxidant status against development of cisplatin-induced renal injury.

### 2.4. STS Therapy Increases Renal H_2_S Content to Activate Renal Arginine/cAMP and NO/cGMP Pathways and Downstream Mechanisms After Cisplatin Administration

**Figure 5:**
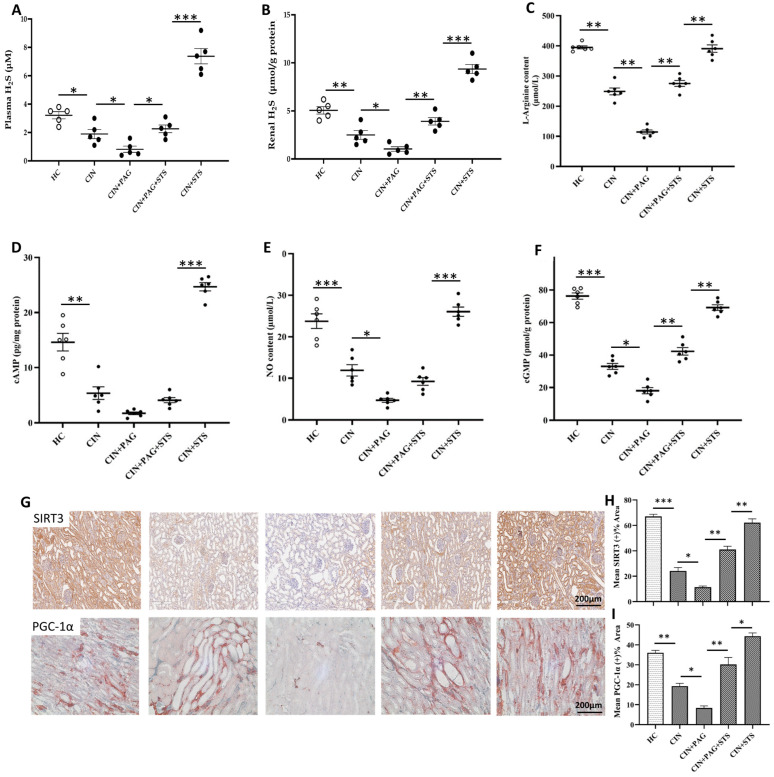
Mechanism of STS chemoprotection. (**A**) Plasma H_2_S level, (**B**) renal H_2_S content, (**C**) plasma arginine level, (**D**) renal cyclic adenosine monophosphate (cAMP) content, (**E**) plasma nitric oxide (NO) level, and (**F**) renal cyclic guanosine monophosphate (cGMP) content. Immunohistochemical staining (**G**), and quantification of (**H**) SIRT3 and (**I**) PGC-1α. HC = Healthy control group; CIN = Cisplatin group; CIN + PAG = Cisplatin group treated with propargylglycine; CIN + PAG + STS = Cisplatin group treated with propargylglycine and sodium thiosulfate; CIN + STS = Cisplatin group treated with sodium thiosulfate. All images were taken at 40× magnification. Values are mean ± SEM. * *p* < 0.05, ** *p* < 0.01, *** *p* < 0.001.

To investigate the mechanism underlying the observed chemoprotective action of STS, we measured plasma and renal content of H_2_S, L-arginine, nitric oxide (NO), cyclic adenosine monophosphate (cAMP) and cyclic guanosine monophosphate (cGMP), as well as their downstream effects (silent information regulator type-3 (SIRT3) and peroxisome proliferator-activated receptor-gamma coactivator-1 alpha (PGC-1α)). In comparison with HC rats, plasma and renal content of H_2_S dropped significantly in CIN rats (Figure 5A,B; *p* < 0.05), with a further drop in CIN + PAG rats relative to the CIN group of rats (Figure 5A,B; *p* < 0.05). However, H_2_S content markedly increased in CIN + PAG + STS group compared to CIN + PAG rats (Figure 5A,B; *p* < 0.05), and increased substantially in CIN + STS rats to a level above that in the HC group (Figure 5A,B; *p* < 0.01). Plasma and renal content of L-arginine, NO, cAMP and cGMP and renal expression of SIRT3 and PGC-1α proteins followed the same trend as H_2_S content (Figure 5C–I). This result shows that STS therapy activates renal production of endogenous H_2_S, which in turn activates arginine/cAMP and NO/cGMP pathways and downstream mechanisms against renal injury caused by cisplatin.

## 3. Discussion

Cisplatin is one of the most widely known antineoplastic agents for a repertoire of human malignancies. The present study investigated CIN as a major side effect of cisplatin administration, and STS, a clinically approved H_2_S donor drug, for chemoprotection against development of CIN. The current study also investigated the chemoprotective mechanism of STS against CIN. As observed in our study, CIN negatively impacted the glomerular and tubular compartments of the kidney, characterized structurally by glomerular hypertrophy and mesangiolysis, as well as acute tubular necrosis. Our observation supports previous experimental findings, showing that cisplatin impairs the glomerulus and glomerular capillaries, and induces proliferation of capsular epithelial cells [16,17,18,19]. It is well-established that epithelial cells, the main cell type of the renal tubules and most vulnerable to a variety of injury stimuli, such as cisplatin, are the most commonly affected in CIN due to the high expression of cisplatin transporters in epithelial cell membrane, and the fact that the dose-limiting toxicity of cisplatin cannot be accounted for by DNA-adduct formation in this region of the nephron [19,20,21,22,33]. This could also explain our observation of tubular necrosis in CIN.

Interestingly, the structural damage associated with cisplatin administration in the present study was exacerbated following pharmacological inhibition of endogenous H_2_S with PAG. However, by targeting the glomerular and tubular compartments of the kidney, STS therapy preserved renal structural integrity against CIN development as observed in our hematoxylin and eosin stain. This suggests that epithelial cells are the targets of STS in the tubular compartment of the kidney. It is worth noting that H_2_S diffuses readily across cell membranes due to its hydrophobicity, and therefore requires no transporter or receptor. Hence, it is highly plausible that the H_2_S released by STS upon administration, diffuses into the tubular epithelial cells and inhibits cisplatin transporters, thereby preventing cisplatin accumulation and transport into the renal tubular epithelial cells. Given that we also observed glomerular protection by STS against cisplatin-induced glomerular damage, it also implies that H_2_S released by STS permeates the membranes of the four cell types of the glomerulus (endothelial cells, podocytes, mesangial cells and parietal epithelial cells) to protect and preserve glomerular integrity. As we were unable to explore the specific signaling pathway through which STS acts on the targeted cells, future in vitro studies would be required to investigate this assertion. Our finding also suggests that reduced renal H_2_S production below physiological levels mediates CIN development and progression, and that its augmentation either by exogenous administration of H_2_S donors (e.g., STS) or increased endogenous production activates the renal H_2_S system to provide chemoprotection against CIN development.

Functionally, CIN in the present study was evidenced by elevated levels of serum creatinine and blood urea nitrogen, significant albuminuria, increased urine production along with decreased water intake, increased plasma Na^+^ and K^+^ levels, and reduced urine creatinine and creatinine clearance. While cisplatin caused these functional changes in the kidney, pharmacological inhibition of endogenous H_2_S production aggravated these pathological changes. As expected, STS therapy attenuated these changes and increased renal H_2_S level substantially. This suggests that renal H_2_S is crucial in preserving renal function following cisplatin administration. Our observation aligns with previous studies in rat and porcine models, in which intrarenal arterial infusion of sodium sulfide (Na_2_S) and sodium hydrosulfide (NaHS), non-clinically viable H_2_S donors, increased plasma H_2_S level and enhanced Na^+^ and K^+^ excretion [34,35]. Although we did not measure natriuresis and kaliuresis in our study due to technical challenges, our observation of severe hypernatremia and hyperkalemia following cisplatin and further increase after PAG administration implies that cisplatin along with inhibition of H_2_S impairs the transport of these electrolytes across the renal tubular membrane, and that STS potentially preserves the renal tubular integrity and increases the excretion of excess of these electrolytes through increased renal H_2_S production. Considering that the kidney regulates blood volume, fluid balance, and blood pressure, and maintains physiological pH, it is important to point out that the severe hypernatremia and hyperkalemia in our cisplatin- and PAG-treated CIN rats has detrimental effects on renal water handling and blood pressure.

Mechanistically, the decreased plasma Na^+^ and K^+^ levels in our STS-treated rats and the increased natriuresis and kaliuresis by other H_2_S donor compounds in other studies [34,35] suggest that H_2_S has inhibitory effect on the activities of renal tubular transporters, such as Na^+^/K^+^-ATPase and Na^+^-K^+^-2Cl^−^ cotransporter, thus inhibiting their reabsorption and enhancing their excretion. In addition, it is also possible that such inhibitory action of H_2_S could be due to its ability to inhibit the activation and opening of epithelial sodium channels in the distal tubule, and also to target H_2_S-sensitive disulfide bonds, which have been identified in specific growth factor receptors in the renal proximal tubule, such as epidermal growth factor receptor, leading to reduced reabsorption and increased excretion of these ions [36,37,38]. Besides the increased hypernatremia and hyperkalemia in our cisplatin- and PAG-treated CIN rats, we also observed increased diuresis in the face of reduced water intake in these groups of rats, which could be interpreted as a water balance disorder. Interestingly, such diuresis was significantly reduced after STS therapy. The increased diuresis could be due to dysfunctional renal aquaporins (AQPs), a family of transmembrane proteins that serve as water channels and mediate bi-directional flow of water, thereby regulating water flow. Among the renal AQPs that span the entire length of the nephron, AQP2 is the principal regulator of urine concentration. Although measurement of urinary AQPs was beyond the scope of our study, a recent study by Luo and colleagues [39] reported in a mouse model of lithium-induced nephrogenic diabetes insipidus (a water balance disorder) that daily administration of the H_2_S donor, GYY4137, for 7 days upregulated the expression of renal AQP2 in the principal cells of the collecting duct, and thereby improving urinary concentrating ability. In the same study, pharmacological inhibition of H_2_S downregulated AQP2 expression and impaired the urine concentrating ability of the collecting duct [39]. This salutary effect was also observed in a rat model of CIN in which alpha-lipoic acid (ALA), an H_2_S-storage compound, upregulated renal AQP1-3 expression in the cortical and medullary regions of the kidney [40]. The upregulated expression of AQPs by GYY4137 and ALA in these studies could also explain the reduced diuresis in the face of increased water intake in our STS-treated CIN rats. One of the novelties in our experimental study is the use of a clinically approved H_2_S donor drug, which facilitates translational application of our work to the clinical setting, and the finding that STS therapy activates arginine/cAMP and NO/cGMP signaling pathways through increased renal H_2_S to attenuate and prevent cisplatin-induced urine concentrating defects. Our finding also supports the suggestion that arginine/cAMP and NO/cGMP pathways and AQPs, particularly AQP1-3, could represent therapeutic targets in the treatment and/or pharmacological management of CIN, and could possibly be extended to water balance disorders.

Reduced renal blood flow (RBF) and glomerular filtration rate (GFR) are commonly associated with cancer patients receiving cisplatin therapy. This clinical hallmark supports our preclinical observation of reduced renal creatinine clearance in our CIN rats. Previous preclinical studies have shown that administration of non-clinically viable H_2_S donors upregulated renal expression of the H_2_S-producing enzymes, CBS and CSE, resulting in increased RBF and GFR [34,35]. Our experimental result is further supported by the finding that GFR, an index of renal creatinine clearance and the principal measure of renal function, was markedly reduced in heterozygous CBS mice (CBS^+/−^) along with other indicators of renal dysfunction in another disease model, while the reverse was observed in CBS^+/−^ mice receiving H_2_S-suppemented drinking water [41], similar to our STS-treated rats. We recognize that one of the limitations of our work is our inability to measure H_2_S-producing enzymes due to technical challenges. However, a recent clinical study of chronic kidney disease patients revealed significantly lower plasma H_2_S and downregulation of CBS and CSE genes in blood mononuclear cells of these patients, which positively correlated with reduced GFR and disease severity relative to healthy control group [42]. Although no H_2_S donor drug, such as STS, was administered in this clinical study, one could infer from previous preclinical findings, including ours, that STS administration would elevate the plasma H_2_S and mRNA levels of these H_2_S-producing enzymes in the blood mononuclear cells and restore GFR in these patients, and thereby improve their kidney condition.

From a mechanistic perspective, the increased GFR in our STS-treated rats suggests that H_2_S exhibits a dilatory effect on the renal vasculature by reducing renal vascular resistance. This suggestion is very plausible, since H_2_S is a direct activator of adenosine triphosphate-sensitive potassium (K_ATP_) channels (the main vascular target of H_2_S) in different vascular tissues and increases K_ATP_ channel current along with membrane hyperpolarization [43,44]. As noted, an important finding in the present study in relation to renal vasodilation effect of H_2_S is our observation that STS therapy increased renal arginine/cAMP and NO/cGMP signaling pathways, while pharmacological blockade of H_2_S inhibited these pathways. It is known that arginine/cAMP and NO/cGMP pathways are among the most widely studied vasodilatory pathways experimentally, although have not been studied in animal models of CIN. Besides arginine/cAMP and NO/cGMP activation by STS in the present study, we also observed upregulated renal expression of SIRT3 and PGC-1α, which were significantly downregulated in our CIN and PAG-treated CIN rats. SIRT3/PGC-1α is one of the downstream signaling pathways of arginine/cAMP and NO/cGMP activation, which is known to preserve mitochondrial integrity by promoting mitochondrial biogenesis, regulating mitochondrial bioenergetics and inhibiting mitochondrial oxidative damage. Therefore, the chemoprotection by STS, including the increased GFR, along with improvement in other parameters in renal hemodynamics as observed in our study and in previous studies, could be partly attributable to the involvement of arginine/cAMP and NO/cGMP pathways and activation of several downstream mechanisms, including SIRT3/PGC-1α signaling pathway. Also, there is a possibility that the renal vasodilation effect of H_2_S could be due to its interaction with other gaseous signaling molecules [45].

In addition to the structural and functional changes induced by cisplatin, which were prevented by STS therapy in the present study, we also noted that cisplatin impaired renal antioxidant status. The impaired antioxidant status following cisplatin administration is characterized by increased level of malondialdehyde (MDA), and decreased glutathione (GSH) content and superoxide dismutase activity (SOD) in the kidney. MDA is a by-product of lipid peroxidation, and its high level indicates the production of reactive oxygen species (ROS), which destroy cells and tissues through oxidative stress. It is known that mitochondria are the main source of ROS production in the body. Although our study did not include examination of subcellular structures such as the mitochondria, our observation could be explained by the upregulated expression of SIRT3 and PGC-1α proteins in the kidneys of STS-treated CIN rats, as well as previous reports showing that nephrotoxic cisplatin metabolites accumulate in the renal mitochondria and inhibit key enzymes such as complexes I and IV of the mitochondrial electron transport chain, leading to significant increase in ROS generation, impaired energy production system, and substantially decreased activities of mitochondrial antioxidants, such as GSH, glutathione peroxidase, glutathione-*S*-transferase and SOD2 [46,47,48]. The decrease in the levels and activities of these mitochondrial antioxidants and our observation of significantly reduced renal expression of SIRT3 and PGC-1α proteins suggest cisplatin-induced oxidative damage, mitochondrial dysfunction and impaired antioxidant defense system. Considering that inhibition of H_2_S caused further decreases in renal GSH content and SOD activity and markedly downregulated renal SIRT3 and PGC-1α protein expression in the present study, it was not surprising to see an overhaul following STS therapy. This corroborates a report by Fard et al. [49] in a similar CIN rat model, in which NaHS restored the activities of renal antioxidants and significantly reduced ROS-induced oxidative stress, thereby contributing partly to mitigation of CIN. Our result and that of Fard and colleagues [49] suggest that STS (via H_2_S) increases natural antioxidant activity, and further suggest the possibility that STS modulates these mitochondrial antioxidants to prevent the formation and accumulation of nephrotoxic cisplatin metabolites in the mitochondria. More recently, STS was reported to exhibit chemoprotection against CIN development by binding to cisplatin in the kidney and inactivating its ability to mediate DNA-induced cell death and mitochondrial damage [50].

As observed in the present study, renal inflammation also characterized CIN. This was seen in our immunohistochemical stain for ED-1 (macrophage infiltration) along with increased plasma and renal levels of pro-inflammatory cytokines (IL-β, IL-6 and TNF-α). Our result indicates that cisplatin triggers influx of macrophages into the renal tissue to promote inflammation through increased production of these pro-inflammatory cytokines. One of the mechanisms by which cisplatin accumulation in the renal tubular cells causes inflammation is by promoting the binding of TNF-α to its receptors on the surface of the renal tubular cells to trigger a cascade of inflammatory events, including activation of neutrophils to produce more pro-inflammatory cytokines and ROS to cause renal injury [51,52]. It is obvious from the present study that the renal inflammatory effect of cisplatin is partly mediated by significantly reduced renal H_2_S content, since H_2_S inhibition enhanced renal inflammation in our cisplatin-treated rats, while its restoration with STS prevented the inflammation. This implies that inhibition of the pro-inflammatory pathway due to activation of the renal H_2_S system by STS is a potential protective mechanism against the pathogenesis and progression of CIN, as was reported in a similar rat model of CIN [49]. Contrary to our finding, one study reported that pharmacological inhibition of endogenous H_2_S production with PAG did not increase renal expression of macrophages and TNF-α, and hence did not exacerbate CIN [53]. This contradictory finding could be due to differences in experimental design, as the authors administered 5 mg/kg of PAG for 4 days in their cisplatin-treated rats, while we administered a higher dose of PAG (40 mg/kg) for a longer duration (28 days).

Finally, our study also showed that tubulointerstitial lesions associated with CIN are partly due to apoptosis of renal tubular epithelial cells, as observed in our TUNEL stain. As expected, inhibition of H_2_S resulted in significant apoptosis of tubular epithelial cells, while STS therapy attenuated and prevented apoptosis. Our finding corroborates previous rat models of CIN in which NaHS protected renal tubular cells from apoptosis and ameliorated CIN progression [49,54]. Although these studies, including ours, did not probe further into the apoptotic mechanisms, it is possible that the release of H_2_S by STS and other H_2_S donor compounds following their administration, prevented activation of the apoptotic machinery by inhibiting several pro-apoptotic pathways, including the opening of mitochondrial permeability transition pores (MPTPs) to prevent the release of pro-apoptotic factors from the mitochondria into the cytoplasm and nucleus. This notion is premised on the fact that the opening of MPTPs has been implicated in CIN development and progression [55,56,57,58,59] and that H_2_S inhibits MPTP opening in other animal models of human diseases [60,61]. Therefore, future studies should consider investigating the anti-apoptotic mechanism of H_2_S in relation to CIN.

One of the limitations of the present study is the use of male rats without inclusion of female rats. Currently, there are controversies regarding the role of sexual dimorphism in CIN. Some studies suggest less CIN vulnerability in the female sex than in male sex due to effects of hormones (e.g., estrogen), antioxidants and other supplements [62,63,64]. In contrast, other studies, including studies of several tissues from cancer patients, also reported a higher renal clearance of cisplatin in male sex compared to female sex, resulting in significant reduction in platinum concentration and markedly lower levels of serum markers of renal injury, as well as lower mortality in male animals than their female counterparts [65,66,67,68,69]. Interestingly, recent clinical studies also showed that these gender-associated differences are not predictors for CIN [70,71,72]. In relation to H_2_S production, a gender dimorphism study of H_2_S production showed that the activity of CSE and H_2_S content in the cardiovascular system in female sex were significantly higher than in male sex under physiological condition [73]. While this has not been studied in CIN, it will be interesting to investigate whether a similar or a different trend would be observed under pathological conditions, such as CIN. In summary, in the light of the controversy about the role of sexual dimorphism in CIN, further preclinical and clinical investigations are required to clarify this ongoing controversy, as well as its relationship with H_2_S production. Nonetheless, our findings contribute to the growing body of existing literature on the chemoprotective effects of gaseous signaling molecules, such as H_2_S, against CIN development and progression.

## 4. Materials and Methods

### 4.1. Ethical Statement on Animal Experimentation

The animal study protocol was approved by the Institutional Animal Care and Use Committee of the University of Ghana (Protocol Number: UG-IACUC 027/22-23). The animal experimentation was conducted with the 3Rs and in accordance with the approved guidelines by the same Committee.

### 4.2. Animal Description and Care

Twenty-five male Sprague Dawley rats (*Rattus novergicus*, 200 ± 20 g of initial body mass) between 6 and 8 weeks were obtained from Noguchi Memorial Institute for Medical Research, University of Ghana, Legon and housed in cages at the Department of Animal Experimentation at the same Institute at 21–25 °C ambient temperature and relative humidity of 45–55% at a 12:12 h light: dark cycle. They were fed with standard commercial pellet diet (Agricare Ltd., Kumasi, Ashanti Region, Ghana) and tap water ad libitum, and were allowed to acclimatize for 7 days in adherence to guidelines set by the Institutional Animal Care and Use Committee of the University of Ghana.

### 4.3. Animal Grouping and Experimental Procedure

#### 4.3.1. Treatment and Euthanasia

Rats were randomly assigned to the following groups. Group 1: Healthy control (HC; received 10 mL/kg/day of 0.9% saline intraperitoneally for 28 days, [n = 5]). Group 2: Cisplatin untreated group (CIN; received single dose of 7 mg/kg cisplatin intraperitoneally, [n = 5]). Group 3: Cisplatin with DL-propargylglycine (CIN + PAG; received single dose of cisplatin [7 mg/kg] followed by daily intraperitoneal administration of the H_2_S inhibitor, DL-propargylglycine [40 mg/kg] immediately after cisplatin administration for 28 days [n = 5]). Group 4: Cisplatin with DL-propargylglycine and sodium thiosulfate (CIN + PAG + STS; received single dose of cisplatin [7 mg/kg] followed by daily intraperitoneal administration of DL-propargylglycine [40 mg/kg] and sodium thiosulfate [150 µM] immediately after cisplatin administration for 28 days [n = 5]). Group 5: Cisplatin and sodium thiosulfate (CIN + STS; received single dose of cisplatin [7 mg/kg] followed by daily intraperitoneal administration of sodium thiosulfate [150 µM] immediately after cisplatin administration for 28 days [n = 5]). The doses of cisplatin, PAG and STS were determined from previous studies [29,74,75]. Rats in each group were kept in metabolic cages for 24 h on day 0, 14 and 29 after cisplatin administration for urine collection. Rats were provided with food while in the metabolic cages. The metabolic cages are designed such that urine samples are kept free from food particles or other contaminants. Following urine sample collection on day 30 after cisplatin administration, rats were euthanized under ketamine (60 mg/kg)/xylazine (10 mg/kg) anesthesia on day 30 after cisplatin administration. Kidneys were harvested and blood samples were collected via cardiac puncture into EDTA and Eppendorf tubes to prepare plasma and serum samples respectively. The left kidneys were fixed in 10% neutral-buffered formalin for histopathological examination while the right kidneys were snap-frozen and stored in −80 °C for additional analysis. As a sensitive indicator of renal damage, relative kidney weight was calculated by expressing each kidney weight as a percentage of the rat’s body weight.

#### 4.3.2. Plasma/Serum Preparation and Measurement of Renal Function

Serum and plasma samples were prepared following centrifugation of blood samples at 1008 g at 4 °C for 15 min. Parameters of renal function, such as urine and serum creatinine, and BUN were determined as previously reported [76], following the manufacturer’s manual (Mindray BS-200 Biochemistry Auto-analyzer, Shenzhen, China). Plasma and renal levels of pro-inflammatory cytokines (IL-1β, IL-6 and TNF-α) were measured by ELISA using a DuoSet Kit according to the manufacturer’s instructions (Quantikine, R&D Systems, Minneapolis, MN, USA) and as described by Bortolon et al. [77]. The levels of plasma electrolytes, such as Na^+^ and K^+^, were measured with an iSTAT Handheld Blood Analyzer (Abbott Laboratories, Chicago, IL, USA). Albumin levels in urine samples were measured using an automatic chemical analyzer (Dirui, Changchun, China) after urine samples were diluted with distilled water at a ratio of 1:50. Renal clearance of endogenous creatinine was calculated using the standard formula: CL_cr_ = U × V/P, where CL_cr_ is creatinine clearance, U is creatinine level in urine, V is total urine volume collected in 24 h and P is plasma creatinine [78].

#### 4.3.3. Measurement of Renal Glutathione and Superoxide Dismutase Activity and Malondialdehyde Levels

Renal glutathione (GSH) content was measured using a GSH-Glo™ kit from Promega (Madison, WI, USA). Briefly, about 50 mg of frozen-kept kidney tissue were homogenized in 1 mL of ice-cold 0.5% KCl and sonicated for 1 min and then centrifuged at 1008× *g* for 10 min at 4 °C. The supernatants were used together with GSH standard in the test kit. Renal GSH content was quantified by chemiluminescence in a SpectraMax 2 plate reader (Avantor, Radnor, PA, USA). To measure superoxide dismutase activity in kidney tissue, the same amount of frozen-kept kidney tissue was used, as previously described method by Xu et al. [79]. Measurement of renal tissue malondialdehyde (MDA, a by-product of lipid peroxidation and indicator of ROS production) was measured as previously described [80].

#### 4.3.4. Measurement of Plasma and Renal H_2_S Content

Plasma and renal H_2_S concentrations were determined as previously described [29]. In brief, H_2_S concentration in plasma was measured using a sulfide ion selective electrode and a sulfide antioxidant buffer prepared from 25 g of sodium salicylate, 6.5 g of ascorbic acid and 8.5 g of sodium hydroxide in 100 mL of distilled water (pH ≥ 13). Renal H_2_S concentration was determined following homogenization of frozen-kept kidney tissue in cold KHPO4 buffer (1:10) and centrifugation at 1008× *g* for 15 min at 4 °C. The supernatant (200 μL) was mixed with 200 μL of 2 mM pyridoxal 5-phosphate, 200 μL of 10 mM L-cysteine and 200 μL of 10% trichloroacetic acid, and incubated for 30 min at 37 °C. Next, zinc acetate (1% *w*/*v*, 250 μL 1%) was added to the mixture to trap H_2_S. Following this, N,N-dimethyl-p-phenylenediamine sulfate (20 mM, 133 μL) in 7.2 M HCl and FeCl_3_ (30 mM, 133 μL) in 1.2 M HCl was added to the mixture. The solution was then transferred to a 96-well plate and absorbance was determined spectrophotometrically at a wavelength of 670 nm. The H_2_S concentration was calculated against a standard curve of NaHS (0.1–100 μM).

#### 4.3.5. Measurement of Plasma and Renal NO, L-Arginine, cAMP and cGMP Content

Nitric oxide (NO) in plasma was determined by Griess method using a total nitric oxide and nitrate/nitrite calorimetric assay kit (Abcam, Toronto, ON, Canada) and as previously described [81]. L-arginine content in plasma was measured using L-arginine assay kit in accordance with the manufacturer’s guide (Abcam, Toronto, ON, Canada). Renal content of cAMP and cGMP was measured by ELISA (Thermo Fisher Scientific, Hillsboro, OR, USA). Concisely, frozen-kept kidney tissues were homogenized in PBS solution at pH 7.4 containing 1 g/mL leupeptin, 1% Triton X-100, 10 g/mL aprotinin, and 1 mM phenyl-methyl-sulfonyl fluoride. Following homogenization, the samples were centrifuged at 1372× *g* at 4 °C for 15 min. A volume of 1.5 mL of the supernatants was pipetted into 2 mL Eppendorf tubes and assayed using cAMP and cGMP ELISA kits, following the manufacturer’s manuals (BioVenic, Hauppauge, NY, USA).

#### 4.3.6. Histopathology and Immunohistochemical Staining

Histological analysis of the kidney was performed as previously described [29]. In a nutshell, paraffin-embedded kidney section was cut at 4 µm thick from similar site (renal cortex) of the tissue and in similar plane, after which the sections were dewaxed and stained with hematoxylin and eosin (H and E) and Terminal deoxynucleotidyl transferase dUTP nick end labeling (TUNEL). The H and E sections were scored blindly and semi-quantitatively under a light microscope (Leica DM1000, Leica Microsystems, Morrisville, NC, USA) by two independent renal pathologists and quantified on the basis of acute tubular necrosis (tubular dilatation, atrophy of epithelial cells and widening of tubular lumen) and glomerular injury (glomerular hypertrophy and mesangiolysis) with 0–5 grading scale: 0 (no observable pathology), 1 (minimal), 2 (mild), 3 (moderate), 4 (marked), and 5 (severe) [75,80].

To determine the degree of CIN and protection by STS, kidney sections were stained for kidney injury molecule (KIM-1; diluted 1:50, Santa Cruz Biotechnology, Dallas, TX, USA), a marker for early renal tubular damage, and ED-1 (diluted 1:500, Serotec Ltd., Oxford, UK), a marker for macrophages, SIRT3 (1:100, Proteintech Group, Inc, Rosemont, IL, USA), a marker for mitochondrial homeostasis, and PGC-1α (1:500, Novus Biologicals, Toronto, ON, Canada), a marker for mitochondrial biogenesis. The stained sections of KIM-1, ED-1 and TUNEL were viewed under a light microscope (Leica DM1000, Leica Microsystems, Morrisville, NC, USA) and 20 fields per section were quantified blindly using ImageJ software version 1.52 (National Institute of Health, USA). Glomerular size was also quantified using the same ImageJ software.

### 4.4. Statistical Analysis

Results were presented as mean ± standard error of the mean (SEM). One-way Analysis of Variance (ANOVA) followed by Tukey’s post-hoc test was used for detecting statistical significance between different groups. Statistical significance between values was considered at probability less than 5%. Statistical analysis was carried out by Prism software, version 8 (GraphPad Software Inc., La Jolla, CA, USA).

## 5. Conclusions

In conclusion, we have provided the first laboratory evidence that draws an association between CIN development, H_2_S action through STS therapy, and arginine/cAMP and NO/cGMP pathways in the kidney. We have demonstrated that STS therapy provides chemoprotection against cisplatin-induced glomerular and tubulointerstitial lesions and renal dysfunction by activating renal arginine/cAMP and NO/cGMP pathways, including their downstream mechanisms involving SIRT3/PGC-1α pathway through increased renal H_2_S production. STS therapy attenuated and prevented CIN development and progression by inhibiting inflammation and apoptosis of renal tubular epithelial cells, while improving antioxidant status and preserving renal function. While further investigations are required to explore other mechanisms underlying STS chemoprotection, our result adds to the emerging knowledge of the use of STS as a chemoprotective drug against CIN. Given that we used an H_2_S donor drug which is already in clinical use, our study is the first to demonstrate that a clinically viable H_2_S donor drug activates renal arginine/cAMP and NO/cGMP pathways and their downstream protective mechanisms to ameliorate and prevent CIN development, and thus provides a molecular basis of STS chemoprotection in cancer patients receiving cisplatin chemotherapy.

## Data Availability

The original contributions presented in this study are included in the article.
